# Distribution and Clinical Significance of Th17 Cells in the Tumor Microenvironment and Peripheral Blood of Pancreatic Cancer Patients

**DOI:** 10.3390/ijms12117424

**Published:** 2011-10-28

**Authors:** Songbing He, Min Fei, Yugang Wu, Dingcheng Zheng, Daiwei Wan, Liang Wang, Dechun Li

**Affiliations:** 1 Department of General Surgery, The First Affiliated Hospital of Soochow University, Suzhou 215006, China; E-Mails: hesongbing1@yahoo.com.cn (S.H.); dingcheng1984@163.com (D.Z.); dv1988114@126.com (D.W.); 2 Jiangsu Institute of Hematology, The First Affiliated Hospital of Soochow University, Suzhou 215006, China; E-Mail: quishi2006@yahoo.com.cn; 3 Institute of Medical Biotechnology, Soochow University, Suzhou 215006, China; 4 Department of General Surgery, The Third Affiliated Hospital of Soochow University, Changzhou 212000, China; E-Mail: wuyugang89@163.com

**Keywords:** pancreatic cancer, Th17 cell, IL-17, tumor microenvironment, angiogenesis, prognosis

## Abstract

This study was designed to investigate the distribution of Th17 cells in the tumor microenvironment and peripheral blood of pancreatic cancer patients, its clinical significance, and the expression profile of Th17 cell-associated cytokines. The percentage of Th17 cells detected by flow cytometry analysis (FACS) was significantly higher in 46 pancreatic tumor tissues (5.28 ± 1.65%) compared with corresponding adjacent normal tissues (2.57 ± 0.83%) (*P* = 0.031). In addition, the percentage of Th17 cells was significantly higher in stage III-IV tumors than stage I-II tumors (*P* = 0.039). The percentage of Th17 cells in peripheral blood of 20 pancreatic cancer patients (3.99 ± 1.15%) was significantly higher than 15 healthy volunteers (1.98 ± 0.57%) (*P* = 0.027). Immunohistochemistry (IHC) was performed to detect IL-17^+^ cells in 46 pancreatic tumor tissues, as well as expression of CD34 in 24 tumor tissues. IL-17 was shown to mainly locate in cytoplasm, and the frequency of IL-17^+^ cells in tumor tissues (39/46) was higher than control (29/46). The presence of IL-17^+^ cells in tumor tissues was associated with tumor, node, and metastasis (TNM) stage, and lymph node metastasis (*P* = 0.012 and *P* = 0.009) but not with patient sex, age, tumor size, and histological grade (*P* > 0.05). Interestingly, distribution of Th17 cells in tumor tissues was positively correlated with microvessel density (MVD) (*r* = 0.86, *P* = 0.018). Furthermore, the median survival time of patients with high and low level of IL-17^+^ cells frequency was 14.5 and 18.5 months respectively (*P* = 0.023). The serum levels of Th17 cell-associated cytokines, IL-17 and IL-23 in 20 pancreatic patients detected by enzyme-linked immunosorbent assay (ELISA) were 69.2 ± 28.5 pg/mL and 266.5 ± 98.1 pg/mL, respectively, which were significantly higher than 15 healthy volunteers (*P* = 0.015 and *P* = 0.02). Moreover, levels of IL-17 and IL-23 were significantly higher in stage III-IV tumors than stage I-II tumors (*P* = 0.04 and *P* = 0.036). This study suggests that increase in Th17 cells frequency and its related cytokines levels in pancreatic tumor tissues may indicate involvement in the invasion and metastasis of pancreatic cancer, which may thereby affect patient prognosis. Therefore, Th17 cells and related cytokines may be served as important immune indicators for predicting the prognosis of pancreatic cancer patients.

## 1. Introduction

Pancreatic cancer is a gastrointestinal malignancy that is a serious threat to human health. The 5-year survival rate of pancreatic cancer is less than 5%, giving pancreatic cancer patients the poorest prognosis among the malignant cancers [1,2]. Pancreatic cancer is often difficult to diagnose in the early stages; only 10–15% of patients are eligible for surgical treatment at diagnosis. Current clinical treatments for pancreatic cancer have limited efficacy, highlighting the need for improved treatment strategies to prolong patient survival [3,4].

Therapies targeting the immune system may represent a promising strategy for the treatment of pancreatic cancer [5,6]. Recently, the role of immune mechanisms in pancreatic cancer has led to the development of novel immune therapeutic targets that have become the focus for pancreatic cancer treatment.

Tumor cells can proliferate indefinitely, evade apoptosis, promote angiogenesis, invade tissues, and metastasize. The non-tumor components in the tumor microenvironment can participate in regulating these complicated processes [7,8]. Immune cells are one of the most important components of the tumor microenvironment [9]. Substantial evidence indicates that the abundance of tumor infiltrating lymphocytes in the microenvironment of certain tumor types is associated with the prognosis of cancer patients, and CD4^+^ T cells play a central role in regulating the immune response through their capacity to coordinate the functions of other immune cell types. Newly discovered CD4^+^IL-17^+^ cells, also named Th17 cells, are an effector CD4^+^T cell subset distinct from Th1, Th2 and Treg cells [10,11]. Th17 cells can produce the cytokine IL-17, which has been shown to play a key role in the pathogenesis of autoimmune disorders and infectious diseases [12–14]. An expanding body of studies indicates that Th17 cells are present at tumor sites such as ovarian cancer and prostate cancer [15,16]. However, the distribution and function of Th17 cells in the pancreatic tumor microenvironment has not been well characterized.

In our research, we analyzed the distribution of Th17 cells in pancreatic tumor tissues and peripheral blood of patients and evaluated the expression profile of related cytokines. We further explored the correlation between the distribution of Th17 cells in the tumor microenvironment and tumor angiogenesis, clinical pathological characteristics and patient prognosis.

## 2. Results and Discussion

### 2.1. Distribution of Th17 Cells in Pancreatic Tumor Tissues as Detected by Flow Cytometry Analysis (FACS)

**Figure 1:**
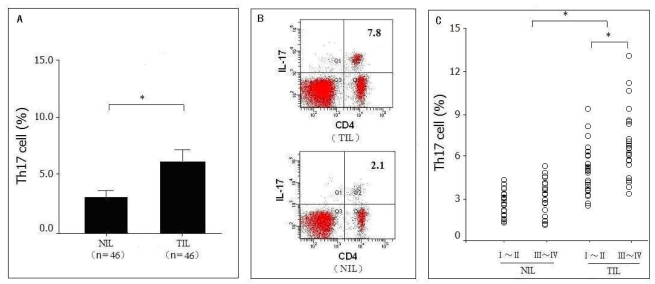
Distribution of Th17 cells in pancreatic tumor tissues. (**A**) Percentage of Th17 cells in TIL (*n* = 46) of pancreatic tumors and NIL (*n* = 46) of corresponding adjacent normal tissues, * *P* = 0.031; (**B**) Representative FACS data from the percentage of Th17 cells in TIL and NIL; (**C**) Percentage of Th17 cells in TIL and NIL of different stages (stage I–II: *n* = 22, stage III–IV: *n* = 24) of pancreatic tumors, * *P* = 0.039.

The expressions of IL-17 in tumor infiltrating lymphocytes (TIL) and non-tumor infiltrating lymphocytes (NIL) were positive, as detected by FACS. However, CD4^+^ cells expressed the majority of IL-17, and CD8^+^ cells expressed little IL-17 in lymphocytes. The frequency of Th17 cells was 5.28 ± 1.65% in the 46 pancreatic tumor tissues which was significantly higher (*P* = 0.031) than in the corresponding adjacent normal tissues (2.57 ± 0.83%) (Figure 1A, B). Moreover, the frequency of Th17 cells in pancreatic tumors of stages III-IV (5.63 ± 1.71%) was significantly higher (*P* = 0.039) than those of stages I-II (3.69 ± 1.40%) (Figure 1C).

### 2.2. Distribution of Th17 Cells in the Peripheral Blood of Pancreatic Cancer Patients as Detected by FACS

**Figure 2:**
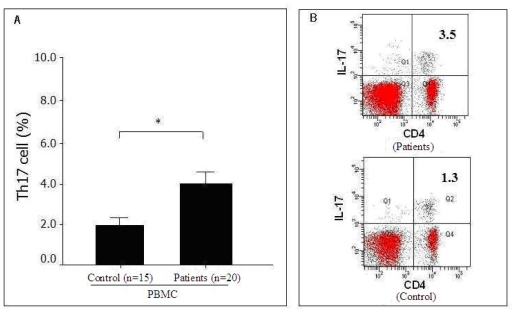
Distribution of Th17 cells in the peripheral blood of pancreatic cancer patients; (**A**) Percentage of Th17 cells in the peripheral blood of pancreatic cancer patients (*n* = 20) and in controls (*n* = 15), * *P =* 0.027; (**B**) Representative FACS data from the percentage of Th17 cells in the peripheral blood of pancreatic cancer patients and in controls.

We also evaluated the presence of Th17 cells in the peripheral blood of 20 pancreatic cancer patients and 15 healthy volunteers. The frequency of Th17 cells in the peripheral blood of the 20 pancreatic cancer patients (3.99 ± 1.15%) was significantly higher (*P* = 0.027) than in the peripheral blood of the 15 healthy volunteers (1.98 ± 0.57%) (Figure 2A,B). However, the frequency of circulating Th17 cells was significantly lower than in tumor tissues from the same patient (data not shown), which suggests that Th17 cells accumulated in tumor tissues or that Th17 cells may have migrated from the peripheral blood to the tumor tissue in pancreatic cancer patients.

### 2.3. Expression of IL-17 in Pancreatic Tumors of Pancreatic *in Situ* as Detected by Immunohistochemisrty (IHC) and Its Correlation with Clinical Pathological Characteristics

**Figure 3:**
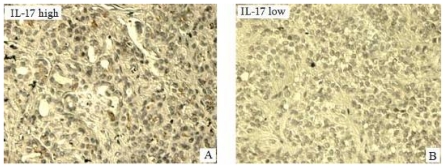
Expression of IL-17 in pancreatic tumors. IL-17^+^ cells stained brown and were present in tumor tissues and in corresponding adjacent normal tissues; (**A**) Representative immunostaining for high distribution of IL-17^+^ cells is shown in pancreatic tumors (original magnification 400×); (**B**) Representative immunostaining for low distribution of IL-17^+^ cells is shown in corresponding adjacent normal tissues (original magnification 400×).

IL-17 was expressed as brown particles distributed in the cytoplasm of tumor cells (Figure 3). Of the 46 pancreatic tumors, 39 were IL-17^+^ (84.8%). However, in corresponding adjacent normal tissues, only 29 out of 46 were IL-17^+^ (63%), although this decrease was not statistically significant (*P* = 0.268). The frequency of IL-17^+^ cells in the 22 stage I-II pancreatic tumors was (3.98 ± 0.45%) which was significantly lower (*P* = 0.012) than in the 24 stage III-IV tumors (5.97 ± 1.25%). The frequency of IL-17^+^ cells in the 35 pancreatic tumors with lymph node metastasis (6.18 ± 1.64%) was significantly higher (*P* = 0.009) than in the 11 patients without lymph node metastasis (3.74 ± 0.55%). Taken together, the frequency of IL-17^+^ cells correlated with TNM stage and lymph node metastasis but did not correlate with sex, age, tumor size and histological stage of the patients (*P* > 0.05) (Table 1).

### 2.4. Expression Levels of Serum IL-17 and IL-23 Detected by Enzyme Immunoassays (ELISA)

**Figure 4:**
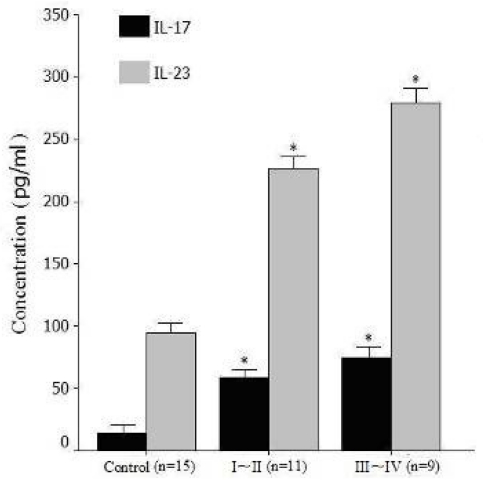
Levels of serum IL-17 and IL-23 in different stages (stage I-II: *n* = 11, stage III-IV: *n* = 9) of pancreatic cancer patients. * *P* = 0.04 and *P* = 0.036, compared with control, respectively.

We also investigated the expression levels of Th17 cell-associated cytokines in serum, serum levels of IL-17 and IL-23 in pancreatic cancer patients were significantly higher than in healthy volunteers (IL-17: 69.2 ± 28.5 pg/mL *vs.* 14.5 ± 6.8 pg/mL, *P* = 0.015; IL-23: 266.5 ± 98.1 pg/mL *vs.* 95.1 ± 37.2 pg/mL, *P* = 0.02). The results are consistent with the increased percentage of Th17 cells in peripheral blood mononuclear cells (PBMC) of pancreatic cancer patients. Moreover, serum levels of IL-17 and IL-23 in stage III-IV patients were significantly higher (*P* = 0.04 and *P* = 0.036) than in stage I-II patients (Table 2, Figure 4).

### 2.5. Correlation between the Distribution of Intratumoral Th17 Cells and Tumor Angiogenesis

**Figure 5:**
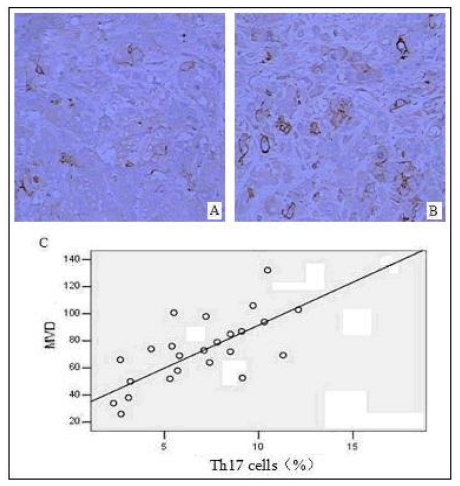
Correlation between distribution of Th17 cells in pancreatic tumor tissues and MVD; (**A**) Representative immunostaining of CD34 in tumor tissues from patients with a low percentage (2.86%) of Th17 cells in TIL (original magnification 200×); (**B**) Representative immunostaining of CD34 in tumor tissues from patients with a high; percentage (9.15%) of Th17 cells in TIL (original magnification 200×); (**C**) Positive correlation between distribution of Th17 cells in pancreatic tumor tissues and MVD (*n* = 24).

Vascular endothelial cells were detected using a mouse anti-human CD34 monoclonal antibody; tumor angiogenesis was evaluated by calculating microvessel density (MVD). Distribution of Th17 cells and was significantly correlated with MVD in 24 pancreatic tumor tissues, as determined by the Pearson correlation analysis (*r* = 0.86, *P* = 0.018) (Figure 5). This suggests that Th17 cells might be involved in tumor angiogenesis and in the promotion of vascular formation of pancreatic tumors, thereby further facilitating tumor proliferation and metastasis.

### 2.6. Correlation between Levels of Intratumoral IL-17^+^ Cells and Survival Time of Pancreatic Cancer Patients

**Figure 6:**
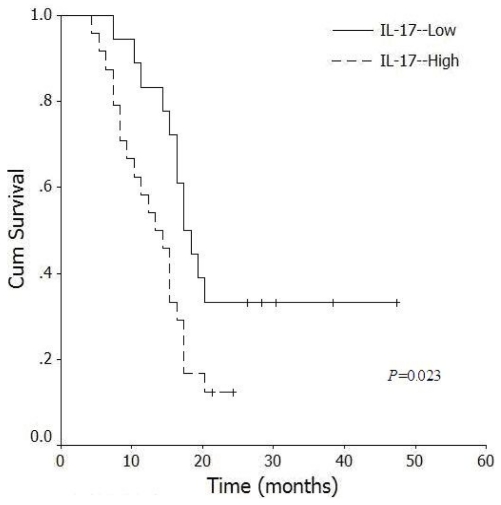
Correlation between levels of IL-17^+^ cells in pancreatic tumor tissues and patient survival (*n* = 46). Survival curves of 46 pancreatic cancer patients with different IL-17 levels are shown. Kaplan-Meier survival curves for high intratumoral expression of IL-17 group were significantly different (*P* = 0.023, log-rank test) from the low expression group.

Of the 46 pancreatic cancer patients, follow up was successful for 42 (91.3%). The follow up period was 5–48 months, and the mean survival time of these patients was 17.5 ± 8.9 months. Patients with higher levels of intratumoral IL-17^+^ cells had significantly shorter (*P* = 0.023) survival time (median, 14.5 months) than patients with lower levels of intratumoral IL-17^+^ cells (median, 18.5 months) (Figure 6). Therefore, an abnormal high frequency of IL-17^+^ cells in pancreatic tumor tissues was correlated with poor prognosis.

### 2.7. Discussion

Upon activation and expansion, naïve CD4^+^ T cells develop into different T cell subsets with different cytokine profiles and distinct effector functions which include Th1, Th2, Treg and Th17 cells. Th17 cells are a recently discovered type of effector T cell [7,8]. The role of Th17 cells in tumorigenesis has become a focus of attention as their function in autoimmune disorders and infectious diseases has gradually been clarified. Both experimental animal models and clinical studies have suggested functions for Th17 cells and its related cytokines in tumor development [14–19], but it is not yet clear for the specific roles of Th17 cells and the mechanism of their involvement in tumor immunity.

To elucidate the roles of Th17 cells and IL-17 in the development of pancreatic cancer, one must first understand the distribution of Th17 cells in pancreatic cancer patients. In our study, we detected Th17 cells in pancreatic tumor tissues by FACS and showed that the frequency of Th17 cells was significantly higher in pancreatic infiltrating lymphocytes than in adjacent infiltrating lymphocytes, which was also associated with tumor stages. Japanese researchers [20] showed that Th17 and Treg cells accumulate in the tumor microenvironment in early stage gastric cancer patients and that the infiltration of Th17 cells decreased gradually as the cancer progressed, whereas the infiltration of Treg cells increased. However, our results were discrepant with this study, which may be due to the different biological characteristics among different tumor types [21]. In addition, the mechanism for regulating the balance of Th17/Treg cells in the tumor microenvironment needs to be further elucidated.

In contrast to our results, it has previously been shown that certain tumors have decreased levels of Th17 cells. The levels of tumor-infiltrating Th17 cells and IL-17 were lowered in the ascites of advanced ovarian cancer patients, which may have a positive effect on the prognosis of the patients [22]. HER2-positive breast cancer patients had decreased levels of Th17 cells compared with HER2-negative patients and with healthy controls [23]. A study on prostate cancer showed that tumor-infiltrating Th17 cells were negatively correlated with the Gleason score [24], which indicates that Th17 cells might result in an anti-tumor immune response. Here, we analyzed the distribution of Th17 cells in the peripheral blood of pancreatic cancer patients and showed that the frequency of Th17 cells was higher in patients than in healthy volunteers. Moreover, the frequency of Th17 cells was significantly lower in peripheral blood than in tumor tissues from the same patient, which suggests that Th17 cells may accumulate in tumor tissues or Th17 cells may migrate from the peripheral blood to the tumor. Therefore, the mechanism of migration and accumulation of Th17 cells into the tumor microenvironment is worth further investigation, as Th17 cells have been shown to be closely associated with certain key cytokines and chemokines [15,18].

IL-17 is a characteristic effector cytokine produced by Th17 cells. IL-17 has six family members (IL-17A to IL-17F), and the IL-17 receptor (IL-17R) family includes five members (IL-17RA to IL-17RE). The biological function of Th17 cells is closely associated with its secreted IL-17 [25,26]. As a member of IL-12 family, IL-23 is the major promoting factor in Th17 cells differentiation and is involved in proliferation and long-term survival of Th17 cells [27]. Using ELISA, we found that levels of IL-17 and IL-23 in the serum of pancreatic cancer patients were significantly higher than in control samples, and these increased levels were associated with tumor stages. Our results suggest that Th17 cells may be involved in pancreatic tumorigenesis through the secretion of associated cytokines.

To further study the function of Th17 cells in pancreatic tumorigenesis, we examined the expression of IL-17 in pancreatic tumors and analyzed the correlation between IL-17^+^ cells and clinical pathological characteristics. We show that a higher frequency of IL-17^+^ cells was correlated with increasing TNM stage and with lymph node metastasis. This suggests that the expression level of IL-17 was closely associated with tumor proliferation and invasion and that abnormal expression of IL-17 can reflect the potential for pancreatic tumor invasion and metastasis. The formation of new tumor vasculature is not only required for tumor growth but is also the primary method of tumor cell invasion and metastasis. We show that the frequency of Th17 cells in tumor tissues was positively correlated with MVD, suggesting that Th17 cells accumulate in tumor tissues and enhance the pro-inflammatory response in the tumor microenvironment, facilitating tumor angiogenesis. As a pro-angiogenic factor, IL-17 has been shown *in vitro* to stimulate vascular endothelial cell migration and cell cord formation as well as to promote tumor angiogenesis, thereby facilitating tumor growth and metastasis [28]. Charles *et al.* [29] showed that TNF-α and IL-17 promote tumor growth synergistically in a mouse ovarian cancer model and in tumor patients. Wang *et al.* [30] showed that IL-17 functions to promote tumors through the IL-6-STAT3 signaling pathway. IL-23 can also stimulate angiogenesis by upregulating the expression of IL-17 and matrix metalloproteinase 9 (MMP9), as well as by decreasing the levels of anti-tumor CD8^+^ T cells in the tumor microenvironment. Therefore, our findings suggest that the accumulation of Th17 cells in pancreatic tumor tissues and the promotion of tumor angiogenesis may be mechanisms of promoting tumor growth, invasion and metastasis. Lastly, to confirm the correlation between levels of IL-17^+^ cells and the prognosis of pancreatic cancer patients, we assessed the survival time of these patients. We showed that increased levels of IL-17^+^ cells were significantly correlated with decreased survival times, which indicates that IL-17 may be a prognostic indicator for pancreatic cancer.

An anti-tumor effect of Th17 cells has been reported, in addition to its pro-tumor effects. In a mouse pancreatic cancer model, Gnerlich *et al.* [31] showed that certain cytokines can regulate the Th17/Treg balance in the tumor microenvironment. Increases in IL-6 in a TGF-β-rich tumor microenvironment can induce the differentiation of CD4^+^T cells to Th17 cells, and increase the IFN-γ ^+^CD8^+^T cell number. Differentiation to Th17 cells inhibited tumor growth and increased the survival rate of mice with pancreatic cancer. Similar studies have shown that anti-tumor immunity can be strengthened by the over-expression of Th1 and Th17 cells in pancreatic cancers, by hybrid vaccines produced by dendritic cells and tumor fusion cells, and by Treg cell depletion [32]. In addition, Th17 cells can induce the production of the Th1-type inflammatory chemokines CXCL9 and CXCL10, recruiting effector cells to the tumor microenvironment which may contribute to anti-tumor immunity. Therefore, it has been speculated that Th17 cells mediate tumor rejection by transforming gradually to Th1-type cells in the tumor microenvironment [33]. However, the specific function of Th17 cells in tumor immunity is still controversial, and it is possible that the function of Th17 cells may vary according to different cancer cause, type, and location, as well as stage of the cancer. Additional studies on the regulation and transformation mechanisms between Th17 cells and other immune cells may elucidate the role of Th17 cells in tumor immunity.

## 3. Materials and Methods

### 3.1. Patients and Specimens

Tissue from 46 pancreatic tumors was collected at the Department of General Surgery, the First Affiliated Hospital of Soochow University. Patients had not received radiotherapy, chemotherapy or immune therapy, and patients with autoimmune or infectious diseases were excluded by preoperative examination. Patient ages ranged from 43–75 years, with the average age at 61 ± 1.5 years old. Patients consisted of 31 males and 16 females. There were 7 stage I, 15 stage II, 18 stage III and 6 stage IV tumors according to the pancreatic cancer TNM stage criteria of American Joint Committee on Cancer (AJCC, 2007). According to histological grade, there were 12 well differentiated, 15 moderately differentiated and 19 poorly differentiated adenocarcinoma cases. The corresponding adjacent normal tissues were used as controls. Fresh specimens were resected under sterile conditions, and two identical portions were prepared. One portion was fixed in 10% neutral formaldehyde, stained with H&E and analyzed by IHC; the other portion was used for FACS. Peripheral blood was collected from 20 out of the 46 patients and 15 healthy volunteers. Collection of all the samples was approved by the Independent Ethics Committee (IEC) of the hospital and was consented to by the patients. Patient survival was assessed through phone calls and clinic visits. Survival time was defined as the period from surgery to last visit or death.

### 3.2. Main Reagents

PC7-labeled mouse anti-human CD4 antibody (Anti-CD4-PC7), PE-labeled mouse anti-human IL-17 antibody (Anti-IL-17-PE) and the Cytofix/Cytoperm kit were purchased from Beckman, USA. Goat anti-human IL-17 antibody and donkey anti-goat IgG were purchased from R&D, USA. Phorbol myristate acetate (PMA), ionomycin and Golgi antagonist Brefeldin A (BFA) were purchased from Sigma, USA. Mouse anti-human CD34 monoclonal antibody was purchased from Beijing Zhongshan Biotechnology Co., Ltd, China. Human IL-17 and IL-23 ELISA kits were purchased from Bender Medsystems, Austria.

### 3.3. Flow Cytometry Analysis

TIL and NIL were isolated from tissue samples by mechanical methods, and PBMC were acquired. PBMC were isolated from the heparinized blood of patients and healthy volunteers. The PBMC were cultured in RPMI 1640 medium that contained 100 U/mL penicillin, 100 U/mL streptomycin and 10% fetal bovine serum. Cell density was adjusted to 2 × 10^6^ c/mL. Cells were stimulated by adding 50 ng/mL PMA, 1 μg/mL ionomycin and 10 μg/mL BFA to the medium for 5 h at 37 °C, 5% CO_2_. Then, 5 μL Anti-CD4-PC7, 100 μL cytoperm and 5 μL Anti-IL-17-PE were added sequentially to the cells. Cells were resuspended in PBS and analyzed by FACS. Th17 levels were defined as the percentage of CD4^+^IL-17^+^ cells in CD4^+^ T cells.

### 3.4. Immunohistochemisrty

The distribution of IL-17^+^ cells and the expression of CD34 in tumor tissues were detected by IHC. Tumor samples were fixed in 10% neutral formaldehyde, embedded in paraffin, sliced and stained with H&E. Briefly, the paraffin-embedded tissues were serially cut into 4 μm sections, dewaxed, and rehydrated. Sections were then blocked with peroxide and non-immune animal serum and incubated sequentially with primary antibody, biotin-labeled secondary antibody, and streptomycin anti-biotin peroxidase. Finally, the sections were stained with DBA, counterstained with hematoxylin, dehydrated, cleared in xylene, and fixed. Known specimens with positive staining were used as positive controls; negative controls were generated by replacing the primary antibody with PBS. To evaluate positive results, 5 fields with condensed IL-17 expression were selected under low magnification (100×) and cell numbers were counted under high magnification (400×). Cells with cytoplasm stained brown were defined as IL-17^+^ cells [17]. Vascular endothelial cells or clusters of brown-stained cells were defined as microvasculature as long as they formed clear boundaries with adjacent capillaries, tumor cells or other connective tissue [34]. The mean value of IL-17^+^ cells and the density of CD34 were calculated in the 5 selected fields. The median value of IL-17^+^ cells was used to categorize the 46 tumor tissues into IL-17^+^ high and low frequency groups [35].

### 3.5. Enzyme Immunoassays

The serum of patients and healthy volunteers were analyzed for the Th17-related cytokines IL-17 and IL-23 using ELISA, following the manufacturer’s instructions. All samples were measured in duplicate.

### 3.6. Statistical Analysis

All data are presented as mean ± SD and were analyzed with SPSS software (version 13.0). Comparisons between groups were performed by Student’s *t*-test. The correlation between Th17 cells in tumor tissues and MVD was determined by Pearson correlation analysis, and the correlation between IHC results and clinical pathological characteristics was determined by Fisher’s exact test. Survival curves were drawn by the Kaplan-Meier method and were compared with the log-rank test. Values of *P* < 0.05 were considered significant.

## 4. Conclusions

In summary, we have identified the levels and distribution of Th17 cells and its associated cytokines in the tumor microenvironment and in the peripheral blood of pancreatic cancer patients. We have analyzed the correlation between levels of IL-17^+^ cells and tumor angiogenesis and with clinical pathological characteristics, and have confirmed that different distribution of IL-17^+^ cells in tumor tissues was closely associated with patient prognosis. Our study has provided an experimental basis for further exploring the mechanisms of Th17 cells in pancreatic tumorigenesis and for establishing an intervention strategy with Th17 cells as a target.

## Figures and Tables

**Table 1:** Correlation between levels of IL-17^+^ cells in pancreatic tumor tissues and clinical pathological characteristics.

Clinic pathological parameters	Cases (*n*)	Frequency of IL-17^+^ cells (%, mean ± SD)	*P* value
**Sex**			
Male	31	4.36 ± 0.85	
Female	15	4.12 ± 0.97	0.408
**Age (years)**			
≤60	17	4.45 ± 0.72	
>60	29	4.78 ± 0.91	0.184
**Tumor size (cm)**			
≤2	32	4.33 ± 0.75	
>2	14	4.91 ± 0.90	0.298
**Histological grade**			
Well differentiated	12	4.35 ± 0.57	
Moderate differentiated	15	4.46 ± 0.71	0.315
Poor differentiated	19	4.70 ± 0.80	
**TNM stage**			
Stage I-II	22	3.98 ± 0.45	
Stage III-IV	24	5.97 ± 1.25	0.012
**Lymph node metastasis**			
Positive	35	6.18 ± 1.64	
Negative	11	3.74 ± 0.55	0.009

**Table 2:** Levels of serum IL-17 and IL-23 in pancreatic cancer patients (pg/mL, mean ± SD).

Group	Cases (*n*)	IL-17	IL-23
Healthy volunteer	15	14.5 ± 6.8	95.1 ± 37.2
Pancreatic cancer patient	20	69.2 ± 28.5 *	266.5 ± 98.1 *
Stage I-II	11	59.1 ± 25.9 *	227.4 ± 87.6 *
Stage III-IV	9	75.8 ± 29.0 *	279.9 ± 102.1 *

* Note: *P* = 0.015 and *P* = 0.02, compared with healthy volunteers.
